# Alcohol Intake and the Risk of Age-Related Cataracts: A Meta-Analysis of Prospective Cohort Studies

**DOI:** 10.1371/journal.pone.0107820

**Published:** 2014-09-19

**Authors:** Wei Wang, Xiulan Zhang

**Affiliations:** Zhongshan Ophthalmic Center, State Key Laboratory of Ophthalmology, Sun Yat-Sen University, Guangzhou, People's Republic of China; University of Missouri-Columbia, United States of America

## Abstract

**Purpose:**

Epidemiologic studies assessing the relationship between alcohol consumption and the risk of age-related cataracts (ARCs) led to inconsistent results. This meta-analysis was performed to fill this gap.

**Methods:**

Eligible studies were identified via computer searches and reviewing the reference lists of these obtained articles. Pooled estimates of the relative risks (RR) and the corresponding 95% confidence Intervals (CI) were calculated using random effects models.

**Results:**

Seven prospective cohort studies involving a total of 119,706 participants were ultimately included in this meta-analysis. Pooled results showed that there is no substantial overall increased risk of ARC due to heavy alcohol consumption. The estimated RRs comparing heavy drinkers versus non-drinkers were 1.25 (95% CI: 1.00, 1.56) for cataract sugery, 1.06 (95% CI: 0.63, 1.81) for cortical cataracts, 1.26 (95% CI: 0.93, 1.73) for nuclear cataracts, and 0.91 (95% CI: 0.32, 2.61) for posterior subcapsular cataracts (PSCs), respectively. No significant associations between moderate alcohol consumption and cataracts were observed. The pooled RRs comparing moderate drinkers versus non-drinkers were 0.90 (95% CI: 0.64, 1.26) for cataract surgery, 0.97 (95% CI: 0.75, 1.25) for cortical cataracts, 0.91 (95% CI: 0.76, 1.08) for nuclear cataracts, and 0.97 (95% CI: 0.49, 1.91) for PSCs, respectively.

**Conclusions:**

This meta-analysis suggests that there is no substantial overall increased risk of ARC due to alcohol intake. Because of the limited number of studies, the findings from our study must be confirmed in future research via well-designed cohort or intervention studies.

## Introduction

Age-Related Cataracts (ARCs) remain the leading cause of blindness in developed and developing countries [1]–[3]. As the world's population ages, visual impairment due to cataracts is on the increase [4]. This is a significant global problem. Although surgical techniques and subsequent outcomes have greatly improved in recent years, the economic cost of cataract surgery remains substantial. Therefore, apart from the surgical extraction of the lens, other primary prevention efforts regarding cataracts should be explored [5].

Alcohol intake may have both harmful and protective effects in terms of ARCs [6]–[9]. An extensive body of data shows concordant J-shaped associations between alcohol intake and a variety of adverse health outcomes [10]. This may be true regarding ARCs. Epidemiologic studies that have assessed this relationship, however, have not consistently shown that heavy alcohol consumption is associated with a higher risk of ARCs or that moderate alcohol consumption is protective [11]–[38]. To address this uncertainty, this meta-analysis of the literature was performed to evaluate the associations between alcohol consumption and ARCs. Only prospective cohort studies were included in this study because of the limitations of retrospective studies in terms of assessing the associations of alcohol consumption due to the significance of recall bias in such studies.

## Methods

The Preferred Reporting Items for Systematic Reviews and Meta-Analyses (PRISMA) statement was used as a guide to conduct the study, including the strategies for searching, analysis, and the presentation of results, potential bias, interpretation, and writing (Checklist S1).

### 1. Literature Search

Two authors independently performed literature searches by using the PubMed and Embase databases through December of 2013. The keywords were as follows: (drinking OR alcohol OR ethanol OR wine OR beer OR liquor OR “life stye”) AND (cataract OR cataracts OR “lens opacity” OR “lens opacities” OR “lens opacification”) AND (cohort OR longitudinal OR inciden* OR follow-up). There were no limits placed on the year or language of publication. References identified from the bibliographies of pertinent articles were also retrieved.

### 2. Exposure Assessment

There are currently no universally accepted definitions of heavy or moderate alcohol use. Low-risk recommendations vary between less than 10 to 60 g/day among developed nations. In this study, heavy alcohol consumption was defined as ≥20 g/day (equivalent to 2 Australian standard drinks) in accordance with the Australian alcohol guidelines average for male and females [39]. Moderate alcohol consumption was defined as being less than heavy alcohol consumption but more than no alcohol consumption. When possible, nondrinkers were chosed as the reference category; however, in several studies, occasional drinkers were included in the reference category. When more than one estimate in a study fell within the range considered for moderate or heavy alcohol consumption, the corresponding estimates were pooled using the Hamling et al. [40] method, thus taking into account their correlation.

### 3. Study Selection

Studies were included for analysis if they met the following criteria: (1) had a prospective cohort study design; (2) clearly reported measurements of alcohol consumption; (3) clearly defined ARC as the outcome; and (4) reported the relative risk (RR) and the corresponding 95% Confidence Interval (95%CI) (or the data to calculate them). In studies of the same population, only the latest or the most complete studies were included. Reviews or letters to the editor without original data, editorials, case reports, case-control studies, and cross-sectional studies were excluded.

### 4. Data Extraction and Study Quality Evaluation

Data extraction and study quality evaluation were performed independently by two reviewers (W.W. and X.Z). Data were extracted using a standardized extraction form, and the assessment of methodological quality was determined by using the Newcastle-Ottawa Scale (NOS) [41]. The NOS consists of three parameters of quality: selection, comparability, and outcome. The NOS assigns a maximum of four points for selection, two points for comparability, and three points for exposure/outcome. A score of nine points on the NOS reflects the highest study quality. Any disagreement was resolved via discussion.

### 5. Statistical Analysis

RR was used as the common measure of association across studies. The data from individual studies were pooled by using the random-effect model with the DerSimonian-Laird method, which considers within-study and between-study variation [42]. Heterogeneity was assessed using the Cochran Q and I^2^ statistics. For the Q statistic, a P value<0.10 was considered statistically significant for heterogeneity; for the I^2^ statistic, values of 25%, 50%, and 75% represented mild, moderate, and severe heterogeneity, respectively. To assess the influence of individual studies on the pooled result, sensitivity analyses were performed by excluding each study one by one and recalculating the combined estimates based on the remaining studies. Subgroup analyses were also performed by gender. Publication bias was evaluated via funnel figures, Egger's test (linear regression method), and Begg's test (rank correlation method) [43]. All analyses were performed by using STATA Version 12.0 (StataCorp). A P value less than 0.05 was considered significant, except where otherwise specified. Additionally, data from this meta-analysis are presented in accordance with the checklist proposed by the Meta-Analysis of Observational Studies in Epidemiology (MOOSE) group.

## Results

### 1. Literature Search

**Figure 1:**
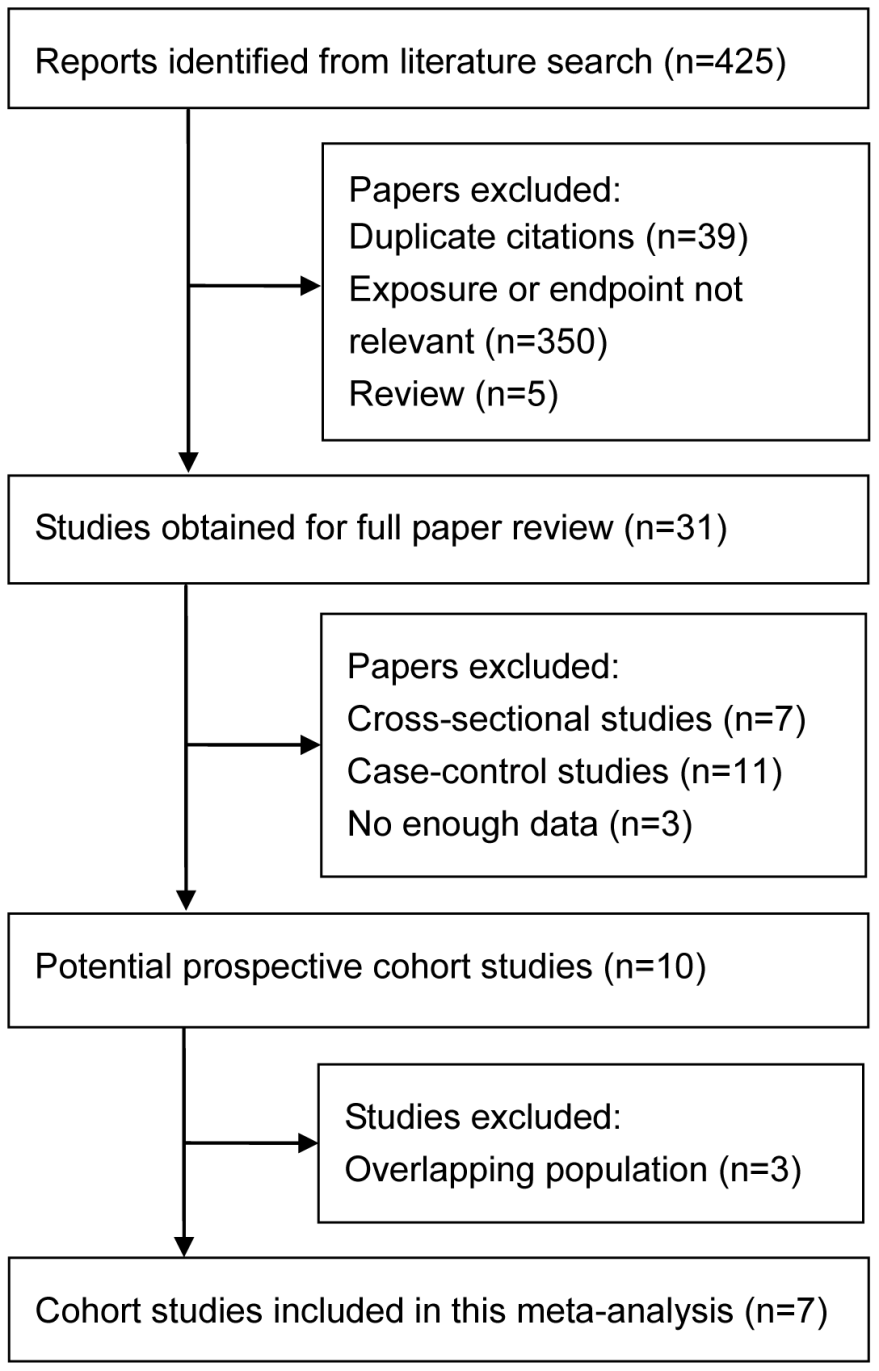
Flow chart of study selection.

The detailed steps of the study selection process are shown in Figure 1. Briefly, we initially identified 425 potentially eligible studies. Thirty-one were considered as potentially relevant studies. Of these, 24 studies were excluded because they did not meet the inclusion criteria or were duplicate publications. Finally, seven cohort studies that met all inclusion criteria were inclued for meta-analysis [29]–[35]. There was complete agreement between reviewers regarding study eligibility.

### 2. Study Characteristics and Qualities

The design features and participant characteristics of the studies are presented in Table 1. The seven prospective cohort studies selected provided a total sample size of 119,706 people. Of these studies, four studies were conducted in North America, one in Sweden, one in Australia, and one in China. The mean length of follow-up ranged from 2 to 12 years, with a median of 8 years. One cohort study included only men, and one study included only women. In most studies, participants were 40 years or older, with the exception of one that also included participants in their 30 s. Five studies were population-based studies, whereas two studies consisted of volunteers (the Physicians' Health Study [35] and the Nurses Health Study [33], which were conducted as part of other studies consisting of healthcare workers). Alcohol consumption was ascertained via self-report at baseline in each study by using self-administered questionnaires that estimated consumption over the past year. The assessment of ARC varied across studies. Standardized criteria for the diagnosis of cataracts were used in some studies, while in others, cases were diagnosed medically via an ophthalmologist or medical record review. Likewise, the outcome measure of cataracts was not consistent. Many studies used the incidence of cataracts, but some studies used cataract extraction as the measure of outcome. All studies adjusted for age in their analyses. The NOS results showed that the average score was 8.5 (range 8 to 9), indicating that the methodological quality was generally good.

**Table 1:** Characteristic of prospective cohort studies evaluating alcohol consumption and its association with age-related cataract.

First author, year	Study Follow-up	Population (Sample Size, age [years])	ARC Definition and Grading	Adjust variables	Alcohol intake	RR(95%CI) (highest vs lowest)
Storey, 2013	The Salisbury Eye Evaluation Study(SEE), 2 years	Population based, United States (2520, 65 to 84)	The Wisconsin Cataract Grading system	Age, sex, race, education, past steroid use, smoking status, alcohol status, history of hypertension, diabetes, average annual ultraviolet-B exposure	Not clear	Any vs non-drink: Nuclear: 0.87(0.63,1.20); Cortical: 1.39 (0.87,2.20); Any: 1.01 (0.78,1.32)
Kuang, 2013	The Shihpai Eye Study (SPES), 7 years	Population based, China (1361, ≥65)	Lens Opacification Classification System III	Age, sex, education, marital status, waist-to-hip ratio, systolic blood pressure, history of hypertension, diabetes, cardiovascular disease, stroke, smoking history, alcohol drinking, and history of hormone use among women	Not clear	Any vs non-drink: Surgery: 1.12(0.64,1.98); PSC: 1.37(0.67,2.82); Any:1.31(0.95,1.81)
Kanthan, 2010	The Blue Mountains Eye Study (BMES), 10 years	Population based, Australia (3654,49 to 97)	The Wisconsin Cataract Grading system	Age, gender, smoking, diabetes, socioeconomic status, steroid use, and myopia.	0 >0 to ≤1 >1 to ≤2 >2 drinks/day	Heavy vs non-drink: Surgery:2.10(1.16, 3.81); Cortical:0.76(0.53, 1.10); Nuclear:1.13(0.73.1.76); PSC: 0.65(0.36, 1.19)
Lindblad, 2007	The Swedish Mammography Cohort (SMC), 8 years	Population based, Sweden, female only (34 713, 49 to 83)	The Swedish National Cataract Register	Age, smoking, alcohol consumption, steroid medication use, vitamin supplement use, educational level	<6 6–13 >13–20 >20–30 >30 g/day	Heavy vs non-drink: Surgery:0.84(0.53,1.33);
Klein, 2003	The Beaver Dam Eye Study (BDES), 10 years	Population based, USA (4926, 43–86)	The Wisconsin Cataract Grading system	Age and Sex	0 >0–5.7>5.7–14.2 >14.2–48 >48 g/day	Heavy vs non-drink: Cortical:1.13 (0.60,2.31); Nuclear:1.93 (1.08,3.46); PSC:0.37 (0.09,1.56)
Chasan-Taber, 2000	The Nurses' Health Study (NHS), 12 years	Nurse, United States (50461, 30 to 55)	Ophthalmologic records	Age, time period, smoking, body mass index, area of residence, number of physician visits, aspirin use, calories, physical activity, parental history of myocardial infarction, history of diagnosis of elevated cholesterol, hypertension, or diabetes	0 >0–4.9 5.0–14.9 15.0–24.9 ≥25 g/day	Heavy vs non-drink: Cataract: 1.10(0.90, 1.35); Cortical: 2.07(0.82, 5.24); Nuclear: 1.10(0.74, 1.62); PSC: 2.46(1.09, 5.55)
Manson, 1994	The Physicians' Health Study (PHS), 5 years	Physician, USA, male only (22071, 40 to 84)	Self-report confirmed by medical record review	Age	Not clear	Daily drinkers vs non: Surgery:1.12 (0.64, 1.98); PSC:1.37 (0.67, 2.82); Any:1.31 (0.95, 1.81)

ARC = age-related cataract; CI =  confidential interval; PSC = posterior subcapsular cataract.

### 3. Alcohol Intake and ARC Risks

**Figure 2:**
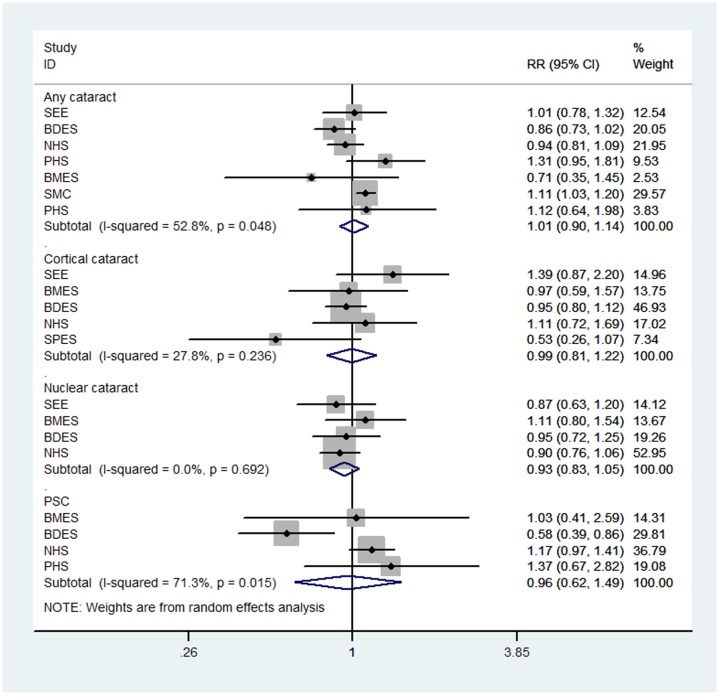
Forest plot for study-specific and pooled relative risk (RR) estimates (Any drinkers versus Non-drinkers) of cataract risk associated with alcohol consumption. SEE = The Salisbury Eye Evaluation Study; BMES = The Blue Mountains Eye Study; BDES = The Beaver Dam Eye Study; NHS = The Nurses' Health Study; SMC =  The Swedish Mammography Cohort; SPES =  The Shihpai Eye Study; PHS =  The Physicians' Health Study; PSC = posterior subcapsular cataract.

The pooled RR of any drinkers versus non-drinkers forARC risk was summarized in Figure 2, and no significant association were noted. There was moderate heterogeneity among studies (P = 0.048; I^2^ = 52.8%). In order to analyse the influence of the sample and age, a sensitivity analysis was performed. When The Nurses' Health Study (NHS) (largest number of population and aged 30–55) was excluded from this study, the random-effect pooled estimate of any drinker vs non-drinker for any cataract was 1.030 (0.893,1.187), similar to that of all 7 studies. Heterogeneity between studies was not significantly reduced by the sensitivity analysis (P = 0.064, I^2^ = 52.0%). In addition, subgroup analyses were performed to evaluate whether the pooled estimates of any drink vs non-drink for any cataract were different according to gender. When NHS study and SMC study were excluded individually from this study, the random-effect pooled RR comparing any drinkers to non-drinkers for any cataract was 0.990 (0.825, 1.188), with moderate levels of heterogeneity (P = 0.174, I^2^ = 37.0%). For the subgroup including studies with only female, the association between alcohol intake and any cataract was also not statistically significant [RR =  1.035 (0.881,1.215)]. However, this should also be interprete with cautions because only two of the seven studies included. These subgroup analyses did not alter the results obtained in cumulative analyses. Hence, the original result was robust.

**Figure 3:**
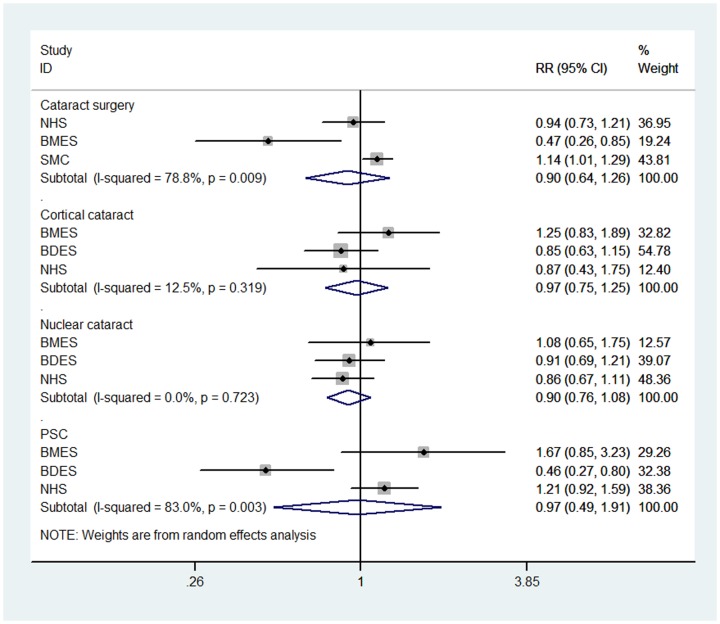
Forest plot for study-specific and pooled relative risk (RR) estimates (Moderate drinkers versus Non-drinkers) of cataract risk associated with alcohol consumption. SEE = The Salisbury Eye Evaluation Study; BMES = The Blue Mountains Eye Study; BDES = The Beaver Dam Eye Study; NHS = The Nurses' Health Study; SMC =  The Swedish Mammography Cohort; SPES =  The Shihpai Eye Study; PHS =  The Physicians' Health Study; PSC = posterior subcapsular cataract.

**Figure 4:**
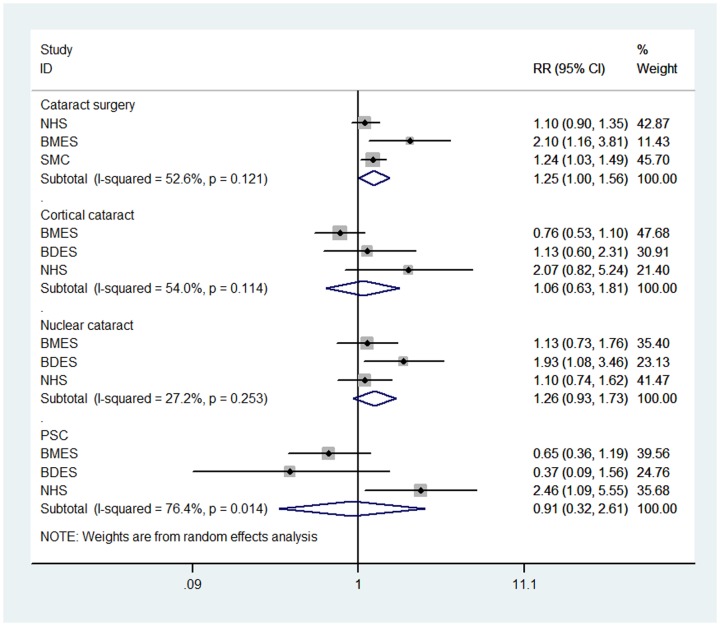
Forest plot for study-specific and pooled relative risk (RR) estimates (Heavy drinkers versus Non-drinkers) of cataract risk associated with alcohol consumption. SEE = The Salisbury Eye Evaluation Study; BMES = The Blue Mountains Eye Study; BDES = The Beaver Dam Eye Study; NHS = The Nurses' Health Study; SMC =  The Swedish Mammography Cohort; SPES =  The Shihpai Eye Study; PHS =  The Physicians' Health Study; PSC = posterior subcapsular cataract.

The RR estimates of moderate drinkers versus non-drinkers were showed in Figure 3. There was also no statistical association. As for heavy drinking versus no drinking, there was a borderline positive association between the risk of cataract surgery and the consumption of ≥20 g/day of alcohol (RR  = 1.25, 95% CI: 1.00, 1.56), with moderate heterogeneity (P = 0.121; I^2^ = 52.60%). With respect to cortical, nuclear, and PS cataracts, the associations were statistically non-significant, with pooled RRs of 1.06 (95% CI: 0.63, 1.81), 1.26 (95% CI: 0.93, 1.73), and 0.91 (95% CI: 0.32, 2.61), respectively (Figure 4). In sensitivity analyses, the results were similar and without great fluctuation (data not shown).

### 4. Publication Bias

**Figure 5:**
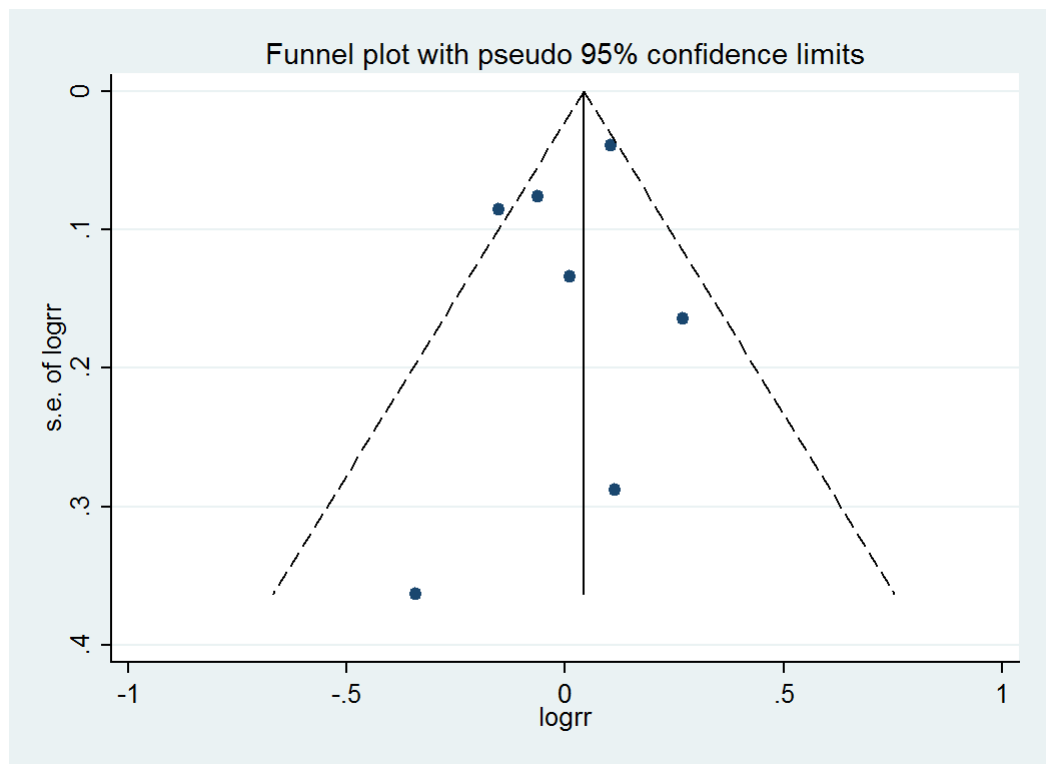
Funnel plot of the association between alcohol intake and risk of any cataract (Any drinkers versus Non-drinkers-Any).

**Figure 6:**
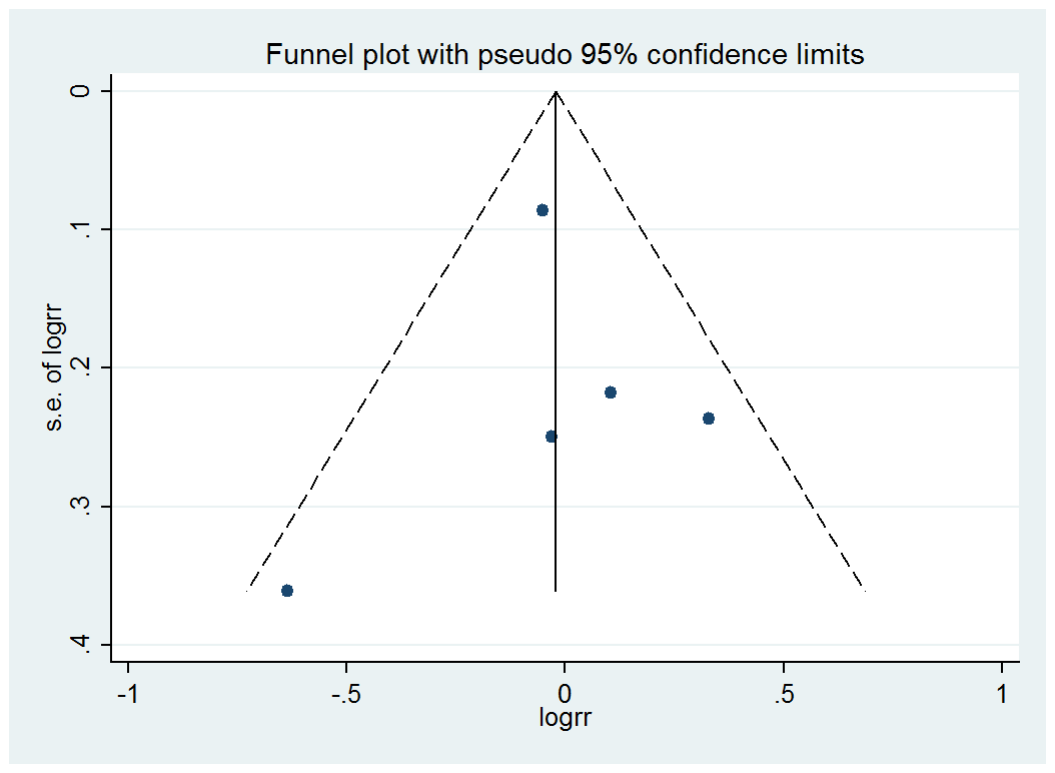
Funnel plot of the association between alcohol intake and risk of cortical cataract (Any drinkers versus Non-drinkers).

Visual inspection of the funnel plot for the studies evaluating alcohol consumption and its associations with ARC did not identify substantial asymmetry (Figure 5, Figure 6). The Begg rank correlation test and Egger linear regression test also indicated little evidence of publication bias among studies of alcohol intake and ARC risk (Begg's Test P = 1.000 and 0.806; Egger's test P = 0.487 and 0.988).

## Discussion

To our knowledge, this is the first meta-analysis to report an association between alcohol consumption and ARC risk. Findings from the current study suggest that there is no substantial increase in the risk of cataract requiring extraction with alcohol intake overall. Similar associations were observed for specific cataract subtypes, however those who reported consuming heavy alcohol had a borderline association with cataract surgery. This combined estimate was robust across sensitivity analyses and had no observed publication bias. However, because of the limited number of studies included, further efforts should be made to confirm these findings.

Alcohol has many metabolic effects and modifies the absorption of drugs and dietary components. These effects may be important in the alcohol-cataract relationship [6]. The lens consists of structural proteins, arranged in a way that allows high transparency. Damaged proteins are eliminated by proteolytic enzymes [44]. With increasing age, the amount of proteolytic enzymes are reduced, thus promoting the formation of protein aggregates, which leads to cataract and loss of visual acuity. Oxidative stress generates free radicals which impair lens proteins, which will aggregate and form opacities [45]. Heavy alcohol consumption induces the microsomal enzyme cytochrome CYP2E1 in the liver. The metabolism of ethanol via this enzyme results in the production of several free radicals. These pro-oxidant molecules generated by the metabolism of alcohol could lead to the aggregation of lens proteins and subsequent cataract formation [46]. In addition, alcohol may augment processes such as membrane damage, alter protein-protein interactions, and disrupt calcium homeostasis [47], all of which contribute to cataract development. However, no substantial association between alcohol consumption and cataract risk was detected in this meta-analysis.

In this study, all RRs were non-significant. With respect to heavy alcohol consumption, the association was borderline significant with RR being 1.25 (1.00 to 1.56). However within the 95% confidence interval is 1.00, hence there may not be significance technically. Furthermore, for studies with small numbers, this study could thus have lacked adequate power to detect possible weak associations between alcohol consumption and the development of individual cataract subtypes. The results indicated that if there is an association between cataracts and alcohol, it is relatively weak. In addition, as individuals vary in their ability to detoxify alcohol in the blood, self-reported consumption of alcohol may not accurately reflect alcohol levels in the body or eye [31], [32]. Therefore, the findings should be interpreted with caution and require confirmation via future studies. In contrast to heavy alcohol consumption, moderate alcohol consumption has been suggested to have a protective effect regarding ARCs. However, we could not confirm this relationship in this study. We were unable to evaluate the shape of the dose-response curve between alcohol consumption and ARCs because of the small number of studies included. Further prospective cohort studies are needed to determine the shape of the dose-response curve. Despite being an attractive factor, our meta-analysis results do not support alcohol intake to have a major effect to ARC susceptibility.

This meta-analysis has several strengths. First, all the original studies used a prospective cohort study design with good study quality, which greatly reduced the likelihood of recall and selection biases. Second, the use of the random-effects model to derive summary estimates allowed the researchers to account for heterogeneity among studies. Moreover, sensitivity analyses were consistent with primary analysis, providing further indication of the robustness of our results.

This study also has a number of limitations. First, although extensive searches with no limitations in terms of language or year of publication were performed, only seven prospective cohort studies evaluating alcohol consumption and ARC were found. Second, because of the inability to fully adjust for various confounders, the adverse effects of alcohol intake on ARCs could be attributed to other confounders related to alcohol consumption, such as poor nutrition, lack of exercise, and high levels of cigarette smoking [29], [30], [35]. However, most included studies have adjusted for a wide range of potential confounders. Third, alcohol consumption measured via self-reporting may be misclassified by study participants due to the social stigma attached to heavy consumption and alcoholism [31]–[34]. In addition, it is possible that heavy drinkers are less likely to participate in studies, which could result in selection biases and also limit the power to detect significant associations with heavy alcohol consumption. Finally, substantial heterogeneity was shown across the component studies. This heterogeneity was not surprising because of variations in the methods of ARC assessment, study designs, and study populations, as well as adjustments across studies.

In conclusion, the present comprehensive meta-analysis provides evidence of a lack of any appreciable association between heavy or moderate alcohol consumption and ARC risk. Because of the limited number of studies, the findings must be confirmed via future research in the form of well-designed cohort or intervention studies. In addition, the underlying mechanisms involved remain to be further elucidated.

## Supporting Information

Checklist S1 **PRISMA checklist.**
(DOC)
